# Identification of bacterial lipo-amino acids: origin of regenerated fatty acid carboxylate from dissociation of lipo-glutamate anion

**DOI:** 10.1007/s00726-021-03109-1

**Published:** 2022-01-25

**Authors:** Amandine Hueber, Yves Gimbert, Geoffrey Langevin, Jean-Marie Galano, Alexandre Guy, Thierry Durand, Nicolas Cenac, Justine Bertrand-Michel, Jean-Claude Tabet

**Affiliations:** 1 MetaboHUB-MetaToul, National Infrastructure of Metabolomics and Fluxomics, 31077 Toulouse, France; 2 IRSD, Université de Toulouse, INSERM, INRA, INPENVT, Université de Toulouse, 3 Paul Sabatier, 31024 Toulouse, France; 3 I2MC, Université de Toulouse, Inserm, Université Toulouse 3 Paul Sabatier, 31432 Toulouse, France; 4 Sorbonne Université, CNRS, Institut Parisien de Chimie Moléculaire (UMR 8232), 4 place Jussieu, 75005 Paris, France; 5 Département de Chimie Moléculaire (UMR 5250), CNRS, Université Grenoble Alpes, 38610 Gières, France; 6 Institut Des Biomolécules Max Mousseron, UMR 5247, CNRS, Université de Montpellier-ENSCM, 34093 Montpellier, France; 7 Université Paris-Saclay, CEA, INRAE, Département Médicaments Et Technologies Pour La Santé, 91191 Gif-sur-Yvette, France

**Keywords:** Lipidomic, Lipoamino acid, Tandem mass spectrometry, Ion–dipole

## Abstract

The identification of bacterial metabolites produced by the microbiota is a key point to understand its role in human health. Among them, lipo-amino acids (LpAA), which are able to cross the epithelial barrier and to act on the host, are poorly identified. Structural elucidation of few of them was performed by high-resolution tandem mass spectrometry based on electrospray combined with selective ion dissociations reach by collision-induced dissociation (CID). The negative ions were used for their advantages of yielding only few fragment ions sufficient to specify each part of LpAA with sensitivity. To find specific processes that help structural assignment, the negative ion dissociations have been scrutinized for an LpAA: the *N*-palmitoyl acyl group linked to glutamic acid (C16Glu). The singular behavior of [C16Glu-H]¯ towards CID showed tenth product ions, eight were described by expected fragment ions. In contrast, instead of the expected product ions due to CONH-CH bond cleavage, an abundant complementary dehydrated glutamic acid and fatty acid anion pair were observed. Specific to glutamic moiety, they were formed by a stepwise dissociation via molecular isomerization through ion–dipole formation prior to dissociation. This complex dissociated by partner splitting either directly or after inter-partner proton transfer. By this pathway, surprising regeneration of deprotonated fatty acid takes place. Such regeneration is comparable to that occurred from dissociation to peptides containing acid amino-acid. Modeling allow to confirm the proposed mechanisms explaining the unexpected behavior of this glutamate conjugate.

**Supplementary Information:**

The online version contains supplementary material available at 10.1007/s00726-021-03109-1.

## Introduction

Among intestinal microbiota metabolites, lipopeptides (Pérez-Berezo et al. 2017) and lipo-amino acids (LpAA) (Vizcaino et al. 2014) have been described for their capacity to regulate host homeostasis. The major technical barrier for the identification of bacterial LpAA or lipopeptides is the difficulty or even the impossibility to isolate a sufficient sample amount to make the analysis either by NMR or/and by X-ray, which would require more than several tens of μg and several mg, respectively. In a previous study, identification of LpAA and lipopeptides produced by *Escherichia coli* Nissle 1917, such as the *N*-lauroyl acyl group linked to asparagine (C12Asn), has been done in negative ESI mode yielding abundant [C12Asn-H]¯ anions (Pérez-Berezo et al. 2017).

Analysis of these metabolites by hyphenated methods as liquid chromatography–tandem high-resolution mass spectrometry LC-HRMS/MS is especially versatile for complex mixture analysis. This method is characterized by a large selectivity, specificity and sensitivity when it is combined to desorption/ionization as electrospray (ESI). Indeed, this mode is suitable for ionization of a broad variety of more or less polar metabolites. Furthermore, it may be a powerful method for structural elucidation of do novo compounds (Thomas et al. 2019) especially, using ion collisional activation (i.e. collision-induced dissociation, CID). First, using low-resolution instrumentation based on linear ion trap quadrupole, an encouraging study of ornithine and glutamine lipids (from *Rhodobacter sphaeroides*) proposed mechanisms (Zhang et al. 2009) or a possible rationalization of ion dissociations under low-energy collision conditions in resonant excitation mode.

In this mode, only the parent ion is excited by its own frequency. Therefore, since the produced ions are not excited, their consecutive dissociations are inhibited unlike competitive dissociations (Bennaceur et al. 2013). However, they occur when the dissociative process is exothermic and/or the residual internal energy carried by the produced ions is sufficient to drive consecutive dissociations (Darii et al. 2021) (Tabet et al. 2021). A previous study (Boukerche et al. 2016) investigated the detected fragment ions of protonated LpAA ([LpAA + H]^+^) resonantly excited under low-energy collisional activation conditions. They interpreted formation of these product ions using exclusively mechanisms based on charge-promoted dissociations. In certain cases, as a consequence, molecular isomerization into ion–dipole as intermediate of stepwise dissociation pathways is considered (Afonso et al. 2010). Such an isomerization explains competitive dissociations mainly based on internal proton transfer into ion/neutral complex responsible of the complementary product ions. This approach is in fact an alternative to the one previously proposed (Zhang et al. 2009) for some fragmentations where the charge remained spectator as it happened during collision process with energies in the keV range (Wysocki et al. 1988; Gross et al. 1992). Interestingly, to test the hypothesis that many lipo-amino acids originate in brain, the identification of new LpAAs was conducted in LC/HRMS/MS by Tan et al. (2010) and explained the observed fragmentations by charge-driven dissociations after CID of selected ions.

With the future aim to identify more metabolites from bacteria of the microbiota and based on these above results, the LpAA characterization will be extended to different amino acids conjugated to various fatty acyl groups using LC-ESI/HRMS/MS. For this, a broader study will be conducted to build a database of product ion spectra (or fragment ion spectra (Murray et al. 2013)) of various LpAA ions with a specific and clear fragmentations of [LpAA-H]¯ to detect, identify and elucidate the do novo molecule structure of LpAA (Thomas et al. 2019). The negative ESI mode was chosen to provide abundant deprotonated LpAA useful to deliver, after CID, fragment ions which are characteristic of both the amino-acid and fatty acyl moieties.

**Scheme 1.**
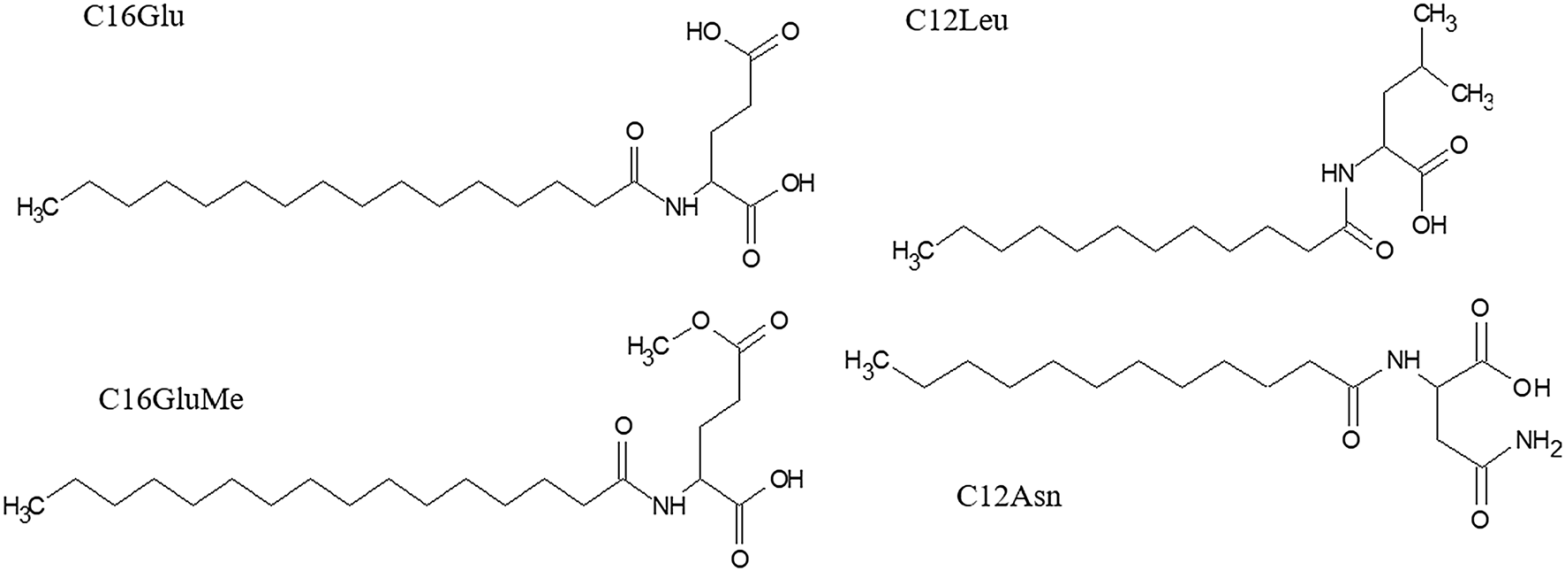
Structure of the studied lipoamino acids: N-palmitoyl acyl group linked to glutamic acid (C16Glu) and monoester derivate (C16GluMe), N-lauroyl acyl group linked to leucine (C12Leu) and to asparagine (C12Asn)

For our purpose, dissociations of deprotonated LpAA standards were investigated under low collision energy conditions (i.e., non-resonant mode) by considering previous studies. In our previous study (Pérez-Berezo et al. 2017), the major dissociative processes of the [C12Asn-H]¯ from N-lauroyl-asparagine occurred, in addition to the water loss (corresponding to the base peak), through competitive covalent bond cleavages around the amide linkage with competitive formations of complementary ion pairs. This takes place through stepwise dissociation via ion/neutral molecular isomerization resulting in the [(C12Asn-H)-(Asn-NH_3_)]¯ and [(Asn-H)-NH_3_]¯ product ion pair through competitive proton transfer allowing to define the two parts of the LpAA. In this study, behavior of the system constituted by both the glutamic residue and the C16 fatty acyl moieties (Scheme 1) was deeply scrutinized. It will be compared to a polar, asparagine (C12Asn) and non-polar amino acid, leucine (C12Leu) (Scheme 1) to reach a pertinent interpretation of the [C16Glu-H]¯ fragmentations based on elemental composition of precursor ion and product ions using an hybrid Qq/TOF tandem mass spectrometer.

Our study will focus on the production of the observed product ions and especially those that are unexpected i.e., product ions generated from LpAA skeleton rearrangement and in particular the carboxylate of the fatty acid. This ion has also been observed and described by Tan et al. (2010). Their work can be considered as a landmark since their proposed interpretation for regeneration of the fatty acid carboxylate from [LpGlu-H]¯. Indeed, without involving the second carboxylic acid group, the proposed tetrahedral intermediate of dissociative [LpGlu-H]¯ evolved directly towards the fatty acid carboxylate product ion. However, dissociation of [LpGABA-H]¯ (containing only one carboxylic acid group) did not lead to regeneration of the carboxylate product ion (Tan et al. 2010). This suggests that the second carboxylic acid in [LpGlu-H]¯ must play important role in the regeneration of the unexpected fatty acid carboxylate product ion. In addition, they suggested that the [(Glu-H)-H_2_O]¯ ion was generated from [Glu-H]¯ by water loss. However, product ion spectrum of [C16Glu-H]¯ (*m*/*z* 384) under resonant excitation conditions displayed, in addition to the H_2_O and CO_2_ losses (ions at *m*/*z* 366 and *m*/*z* 340), only the pair of the deprotonated fatty acid and dehydrated glutamate product ions (Figure S1) which evidences that the latter ions is directly formed from the precursor ion rather than from dehydration of glutamate anion as intermediate.

To rationalize formation of this particular species, the product ion spectrum of the deprotonated [C16GluMe-H]¯ mono ester (Scheme 1) was investigated and compared to [C16Glu-H]¯. For a better description of the origin of the unexpected regeneration of the deprotonated C16 fatty acid, the respective product ion spectra of the C16Glu and C16GluMe anions were investigated under different collision energy conditions. Finally, this study provided an analytical approach to determine without ambiguity the structure of lipo-amino acids and lipopeptides within the framework of mass spectrometry analysis essential for bacterial LpAA identification. For this purpose, it is necessary to better account for the dissociation processes of these compounds by means of well-defined systematic mechanisms. The described mechanisms must explain formation of both the expected and unexpected product ions with similar mechanisms based on the chemical reactivity.

## Materials and method

### Chemical synthesis of LpAA

Synthesis of lipoaminoacids such as *N*-lauroyl-asparagine (C12Asn), *N*-lauroyl-leucine (C12Leu), *N*-palmitoyl-glutamic acid (C16Glu) and *N*-palmitoyl-glutamic acid 5-methyl ester (C16GluMe) were done by solid phase chemistry in a SPS reactor using a 2-chlorotrityl resin (max loading 1.6 mmol/g, 250 mg, 400 µmol, 2.0 eq.). Very briefly, after the fixation of the appropriate Fmoc-amino acid (200 µmol, 1.0 eq.) in presence of NMM (800 µmol, 4.0 eq.) in DCM for 3 h, the resin was capped by DCM/MeOH/NMM (17/2/1) for 5 min and the Fmoc group removed with Piperidine/DMF (2/8) for 10 min. After washing, the fatty acid is added in presence of HBTU (750 µmol, 5.0 eq.), HOBt (15 µmol, 0.1 eq.) and NMM (7.5 mmol, 10.0 eq.) in NMP (5 mL) for 3 h to overnight monitoring with a Kaiser’s test. After washing, the resin is cleaved with TFA/TIPS/Water (95/2.5/2.5) for 10 min. Obtained filtrate were concentrated and triturated in ACN to obtained desired LpAA without further purifications: C16Glu (53 mg, Yield = 69%); C12Leu (112 mg, Yield = 67%); C12-Asn (63 mg, Yield = 100%) C16GluMe (30 mg, Yield = 58%). LpAA were dissolved in methanol with a concentration of 10 ng/µL.

### Mass spectrometry analysis

*Direct infusion mass spectrometry analysis* were done in negative mode ESI(-) using a Xevo G2-XS time of flight (Qq/TOF) mass spectrometer (Waters, Milford, MA) equipped with electrospray ionization (ESI). The source parameters were set as follows: source temperature = 120 °C, capillary voltage = − 2.6 kV, desolvation gas flow rate = 300 L/h, cone gas flow = 40 L/h, desolvation temperature = 380 °C, collision gas nitrogen pressure = 10^–1^ mbar, voltage QqTOF = 9.00 kV, pressure in flying tube = 2.7 × 10^–7^ mbar, average number of spectra = 35 (1 spectra every 14 ms for 500 ms).

*MS/MS experiments* product ion spectra of the precursor [C16Glu-H]ˉ ion were performed in segmented RF only quadrupole (Waters, Milford, MA) under collision-induced-dissociation conditions (i.e., CID, using non-resonant excitation mode) within a full width at half maximum (FWHM) resolution of 16 000 resolution at *m/z* 400 for product ion analysis. Precursor ions were selected within ± 5 *m/z*. The product ion spectra (Murray et al. 2013) (called also CID spectra) were recorded at two collision energies (noted as *E*_Lab_): 17 eV and 30 eV. These CID spectra were inspected manually using MassLynx software (Waters) to confirm annotations (vide infra). Only the signal higher than 5% of base peak were annotated in CID spectra and in the supplementary Table S1, the reported signals were those with relative abundances ≥ 0.4% of the total ionic current TIC at one *E*_lab_ values. The ion abundances were relative to TIC which included precursor and product ions and were given in percent of TIC. Relative and absolute ion abundances are reported in the supplementary Table S1.

### Modeling

DFT calculations were performed using Gaussian16 package, employing the B3LYP functional with the 6–31 + G(d,p) basis set. Zero Point Energies (ZPE) are added as computed at the optimizing level. The nature of all stationary points was confirmed by analyzing the harmonic vibrational frequencies. The energies reported in this work are *E* energies in kcal/mol, corrected from ZPE. For reasons of computational time and under the reasonable assumption that the length of the aliphatic chain will not significantly influence the nature of the pathways studied, the structures were modeled with an ethyl group (for computational details see Supplementary Information S1).

### Nomenclature and notation of product anions

**Scheme 2.**
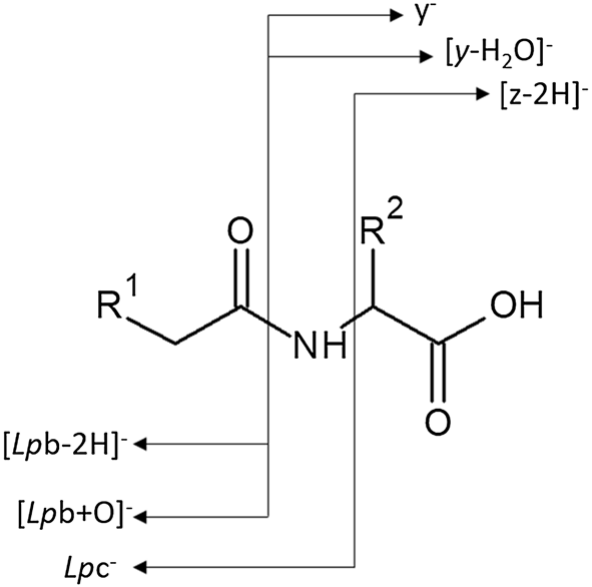
Annotation of the observed product ions from dissociation of deprotonated lipoamino acids

The notations used to describe and discuss mass spectrometry results were based on IUPAC recommendations (Murray et al. 2013) to provide more accurate and consistent text throughout the manuscript. In addition, it was proposed a simple nomenclature which could be applied to the LpAA anion dissociation (Tan et al. 2010). In this nomenclature, only some fragmentations are described and concern only the loss of small neutrals (H_2_O, NH_3_, CO_2_….) which can be also consecutive losses (H_2_O + CO_2_) from the amino-acid (AA) residue. Such an annotation suggests their formations occur through consecutive fragmentations from this AA residue generated by dissociation of LpAA anions. This is not true since some of these fragment ions (the abundant ones) are generated from direct cleavages of the deprotonated lipo-amino acid and not from the amino-acid as intermediate. This nomenclature cannot be applied for deprotonated lipo-peptide dissociations since, *N*-terminus (linked to fatty acyl moiety) or C-terminus (related to peptide moiety) product ions from peptide bond cleavages cannot be attributed. To meet this requirement, the nomenclature on deprotonated peptide dissociations (Chu et al. 2015) based on fragmentations of protonated peptide ions (with (i + j) residues in sequence) yielding was adapted, e.g. fragment ions as [b_i_-2H]ˉ, c_i_ˉ for the N-terminus and y_j_ˉ, [z_j_-2H]ˉ for the C-terminus. This was based on the nomenclature of product ions generated under keV collision energy conditions introduced by Roepstorff et Fohlman (Roepstorff et Fohlman 1984) and Biemann (Biemann 1988). This nomenclature as reported in Scheme 2 allows to describe both the lipo-amino acid and lipo-peptide fragmentations.

Indeed, annotation distinction between (i) fragment ions constituted by the fatty acyl moiety (related to N-terminus of peptide sequence) was annotated by the *Lp* prefix linked to peptide fragment (e.g. [*Lp*b_i_-2H]ˉ, *Lp*c_i_ˉ …) and (ii) fragment ions related to the C-terminus peptide sequence contains exclusively a peptide fragment sequence (e.g. y_j_ˉ, [z_j_-2H]ˉ …). When Glu or Asp are included in the peptide sequence, then a complementary ion pair such as [*Lp*b_i_ + O]ˉ and [y_j_-H_2_O]ˉ appeared. From the lipo-amino acids with AA = Glu, the particular product ions specific to (i) the fatty acyl moiety such as fatty acid amidate and fatty acid carboxylate formally annotated as *lp*c¯ and as [*lp*b + O]ˉ, respectively, and (ii) the amino acid residue such as deprotonated AA (i.e., [Glu-H]¯), and its fragment [(Glu-H)-NH_3_]¯ ion annotated as y¯ and [z-2H]¯ were detected. The consecutive fragment ions due to the small neutral loss, e.g. CO_2_ or H_2_O, from the first generation product y¯ and [z-2H]¯ ions, will be annotated as [y-CO_2_]¯ and [(z-2H)-H_2_O]¯, respectively.

## Result and discussion

**Fig. 1:**
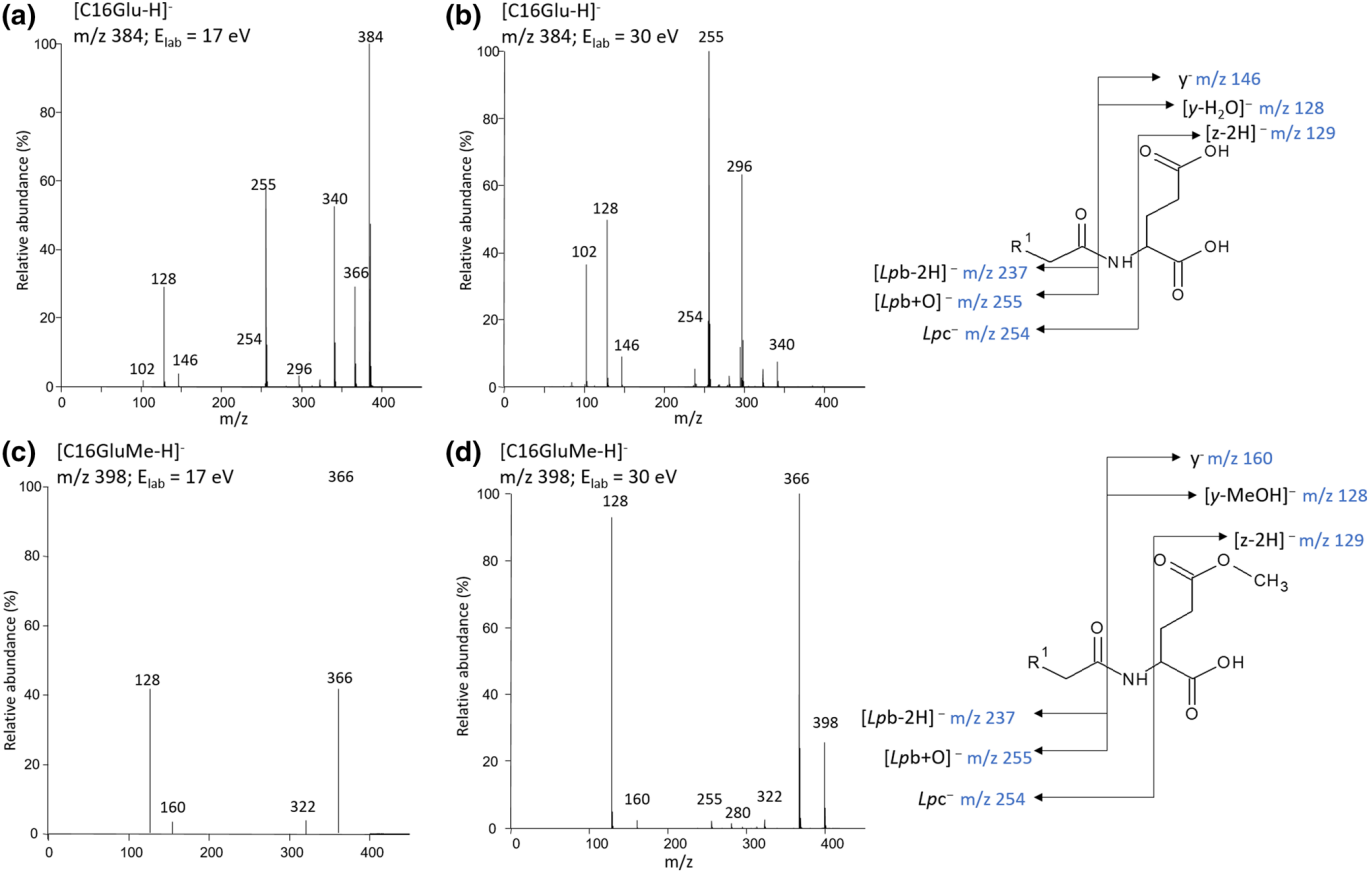
Product ion spectra (i.e., MS^2^ fragmentations) of (a)-(b) [C16Glu-H]ˉ (*m*/*z* 384 with R_1_ = CH_3_(CH_2_)_13_-) at *E*_Lab_ = 17 eV and *E*_Lab_ = 30 eV, and (c)-(d) [C16GluMe-H]ˉ (*m*/*z* 398) at *E*_Lab_ = 17 eV and E_Lab_ = 30 eV, respectively (accurate m/z values and corresponding elemental composition reported in Table S1)

The mass spectrum of the C16Glu compound (Mw 385 u) displayed essentially the [C16Glu-H]ˉ anions (base peak at *m*/*z* 384, Figure S1). Under the source conditions used, adduct ions [C16Glu + anion]¯ (anions as inorganic or organic acids) as well as deprotonated [(C16Glu + Na–H)-H]¯ salt were not observed even if the concentration of C16Glu was increased. This behavior characterized the other LpAA studied. The absence of adduct ions was due to the presence of one or several sufficiently acidic sites able to protonate the reactive anions from the used solvents and allowed to reach a large sensibility of the [C16Glu-H]¯ detection. In MS/MS mode, this is one of the advantages for the investigation of the dissociations of the [C16Glu-H]ˉ anion to perform structural analysis of this family of conjugates. Such a detailed studied were only performed for protonated LpAA (Boukerche et al. 2016) which as expected was driven different dissociation pathways. The [C16Glu-H]ˉ anion (*m*/*z* 384) submitted to collisional activation (CID with *E*_lab_ = 17 eV, and *E*_lab_ = 30 eV, Fig. 1a, b) was characterized by a product ion spectrum (at *E*_lab_ = 17 eV) displaying tenth peaks representative to product ions. The peaks with intensities lower than 5% of the base peak at *E*_lab_ = 17 eV were annotated in the CID spectrum only when their intensities became higher than 5% of base peak at *E*_lab_ = 30 eV, and conversely for the intensities of base peaks lower than 5% at *E*_lab_ = 30 eV and higher than 5% at *E*_lab_ = 17 eV.

In our previous study (Pérez-Berezo et al. 2017), two intense peaks at *m*/*z* 198 (ion *lp*c¯) and *m*/*z* 114 (ion [z-2H]¯) emerged in addition to the base peak at *m*/*z* 295 (related to the H_2_O release) from CID spectrum of [C12Asn-H]¯ (*m*/*z* 313, Figure S2). The complementary *lp*c¯/[z-2H]¯ ion pair were interpreted to be formed via ion/dipole intermediate which allows the production of these complementary fragment ions. By analogy with C12Asn, the product ion spectrum of the [C16Glu-H]ˉ anion (*m*/*z* 384) (Fig. 1a, b) should display the fragment ions at *m*/*z* 254.2483 (ion *lp*c¯ as C_16_H_32_NO with error of 2.5 ppm), and *m*/*z* 129,0193 (ion [z-2H]- as C_5_H_5_O_4_, with error of 151.6 ppm. This product fragment ion *m*/z 129 may match, with the first ^13^C isotopologue of the *m*/*z* 128 ion (ion [(y-H_2_O)-1]- as ^12^C_4_^13^C_1_H_6_NO_3_ with error of 1.7 ppm). Surprisingly, the abundance of this ion *lp*c¯ and [z-2H]¯ pair was of very low. A pair of product ions corresponding to [*lp*c + 1]¯ (at *m*/*z* 255) and [(z-2H)-1]¯ (at *m*/*z* 128) appeared. Less abundant fragment ions corresponding to either small size neutral losses or cleavage of bonds close the amide linkage were displayed in the CID spectra (Fig. 1a, b). The latter corresponded to product ions with charge retention at the amino acid moiety or at the fatty acyl part as confirmed by high-resolution measurements (Table S1 and interpretations were reported in Schemes S1 and S2).

These signals were detected at:

(i) *m*/*z* 366 and *m*/*z* 340 are abundant species accompanied by weaker ion at *m*/*z* 322 (less than 5% of TIC at this energy at *E*_lab_ = 17 eV and increases to 1.8 at 30 eV) and *m*/*z* 296 (~ 1% at 17 eV and 21% at 30 eV), respectively, due to the expected H_2_O, CO_2_, (H_2_O + CO_2_) and 2CO_2_ small size neutral losses (proposed fragmentation mechanisms in Scheme S1);
(ii) *m*/*z* 146 (i.e., y¯) and *m*/*z* 102 i.e., [y-CO_2_]¯, < 1% at 17 eV and 13% at 30 eV) resulting from the loss of fatty acyl moiety (proposed fragmentation mechanisms reported Scheme S2). The detected *m*/*z* 129 ion which was the ^13^C natural isotopologue of the abundant *m*/*z* 128 ion (i.e., [y-H_2_O]¯) and not the [y-NH_3_]¯ isobaric ion, is not reported in Table S1.

**Scheme 3.**
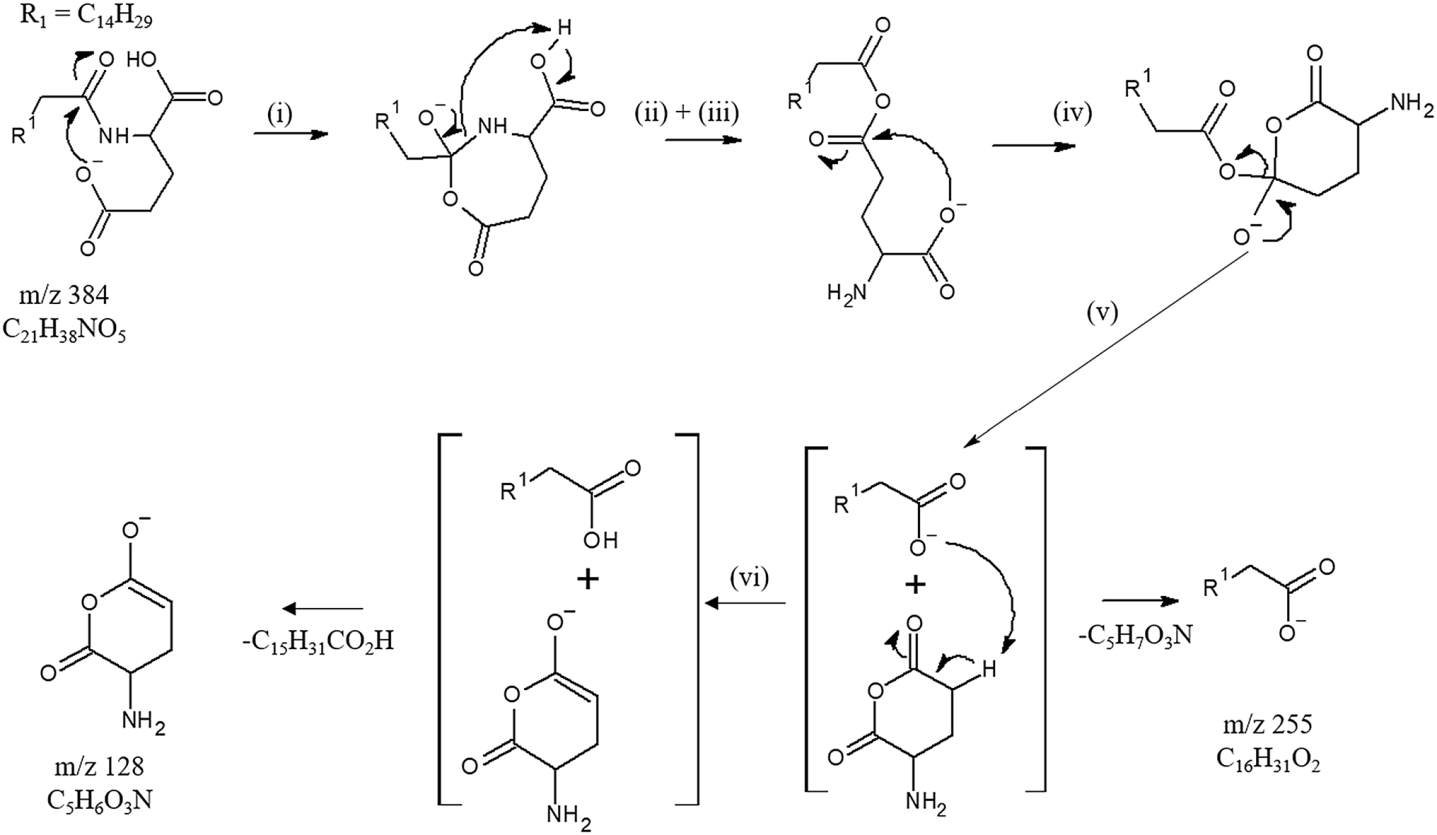
Proposed regioselective stepwise formation of complementary [y-H_2_O]¯ (*m*/*z* 128) and [*lp*b + O]¯ (*m*/*z* 255) product anions through ion–dipole complex molecular isomerization

Since the formation mechanisms of product ions described above have already been studied (Pérez-Berezo et al. 2017), we focused on the origin and formation mechanism of the abundant unexpected *m*/*z* 255 and *m*/*z* 128 product ions displayed in the CID spectra recorded at 17 eV and 30 eV. These complementary ions are considered together rather than separately as proposed by Tan et al. (Tan et al. 2010). The elemental compositions attributed to the ions at *m*/*z* 255.2321 and at *m*/*z* 128.0363 were, respectively, C_16_H_31_O_2_ (error of 3.3 ppm) and C_5_H_6_NO_3_ (error of 2.4 ppm) (Table S1). Thus, they may be, respectively, annotated as the complementary [*lp*b + O]¯ and [y-H_2_O]¯ product ions rather than [*lp*c + 1]¯ and [(z-2H)-1]¯. Indeed, formation of the [y-H_2_O]¯ and [ *lp*b + O]¯ ion pair can be rationalized by a stepwise dissociation process from [C16Glu-H]¯ ion through molecular isomerization into ion/dipole complex intermediate followed by competitive splitting processes (Scheme 3) (Afonso et al. 2010). The first step is a regioselective nucleophilic attack of amino acid side chain carboxylate site to amide linkage resulting in cyclic tetravalent intermediate. The negative alkoxide site migration induces the C–N bond cleavage and ring opening concomitant with proton transfer to nitrogen atom and resulting in an anhydride linkage. The end group carboxylate interacts with the closer anhydride C=O group yielding a 6-membered tetravalent system which isomerizes in ion/dipole consisting in α amino anhydride neutral and long chain carboxylate. This complex can evolve either to a direct partner splitting to yield *m*/*z* 255 (i.e., the fatty acid carboxylate as [*lp*b + O]¯) or after internal proton transfer from the amino anhydride neutral to the C16 carboxylate partner, release fatty acid neutral to drive to the deprotonated 6-membered amino-anhydride (*m*/*z* 128 annotated as [y-H_2_O]¯) (Scheme 3).

The regioselectivity of the first step was confirmed by the CID spectrum of [C16GluMe-H]¯, *m*/*z* 398 for which the peak at *m*/*z* 255 was strongly reduced in this product ion spectrum (Fig. 1c, d). Indeed, the peak at *m*/*z* 255 which was the base peak in the CID spectra of [C16Glu-H]¯ did not reach 1% of base peak at *E*_Lab_ = 17 eV and 9.3% at *E*_Lab_ = 30 eV for [C16GluMe-H]¯. This confirmed that the nucleophilic reactivity of the carboxylic acid in α position of amide linkage is significantly hindered compared to the carboxylic group of the side chain. In addition, the CID spectra of [C12Leu-H]¯ (*m*/*z* 312), a non-polar LpAA, (Scheme S2 and Table S1) did not display peak at *m*/*z* 199 ion corresponding to the [*lp*b + O]¯ ion confirming the very weak reactivity of the amino α carboxyl group. On the other hand, this explained also why the dissociation of the [LpGABA-H]¯ ion did not yield the *lp*c¯ product ion (Tan et al. 2010).

**Fig. 2:**
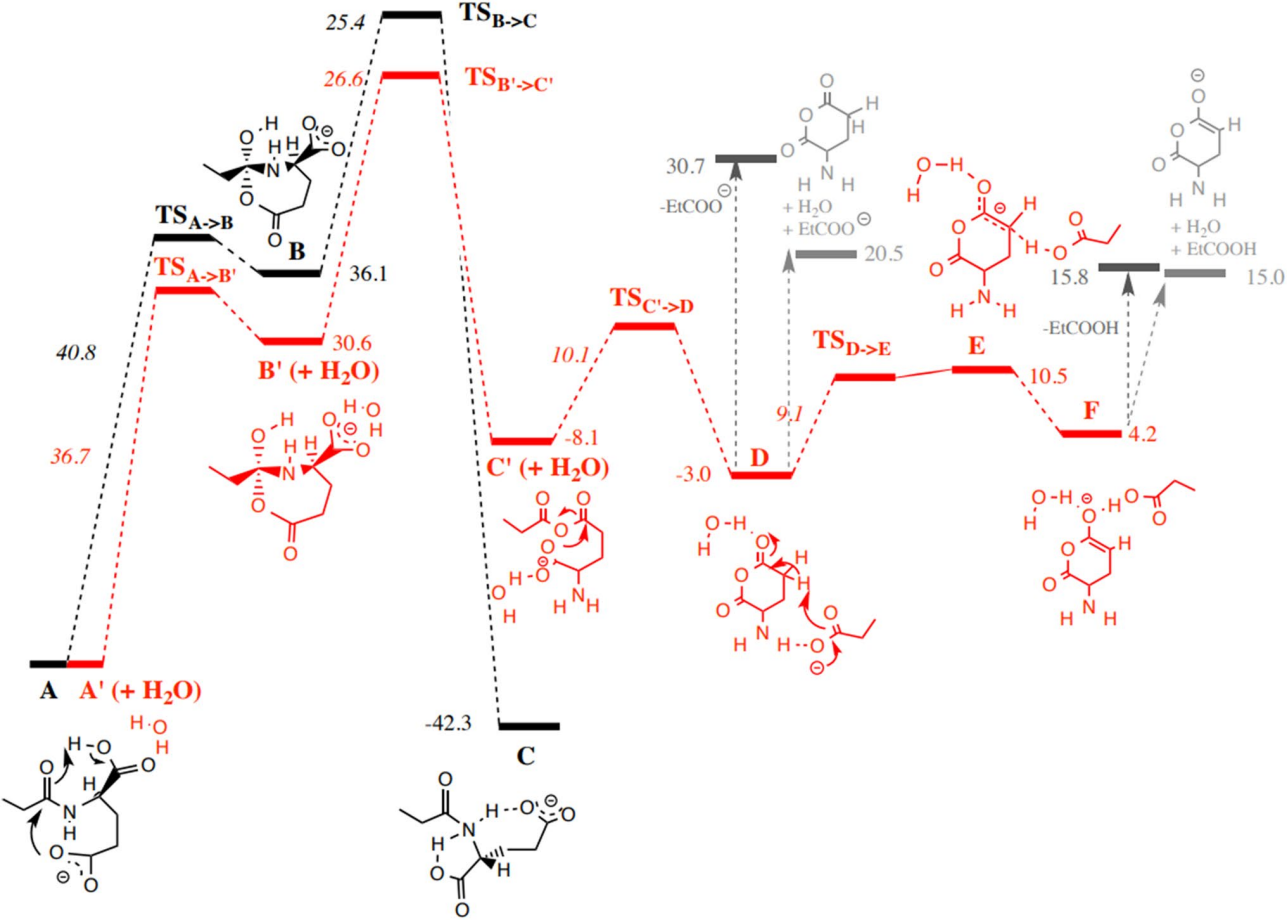
Energetic profiles (B3LYP/6–31 + G(d,p)), ZPE at the optimizing level): **a** the black pathway, an evolutionary dead-end yielding the A carboxylate isomerization into the deprotomer **C** and **b** the red pathway: competitive fragmentations of the hydrated (**A** + H_2_O) carboxylate towards the product anions as (i) propionic carboxylate and (ii) the deprotonated α amino glutaric anhydride (energy in kcal/mol)

To confirm the validity of the proposed mechanism above, modeling was performed. A first investigation was carried out starting from the structure **A** corresponding to the LpGlu (pathway in black, Fig. 2). For reasons of computational time and under the reasonable assumption that the length of the aliphatic chain will not significantly influence the nature of the pathways studied, the structures were modeled with an ethyl group.

**Scheme 4.**
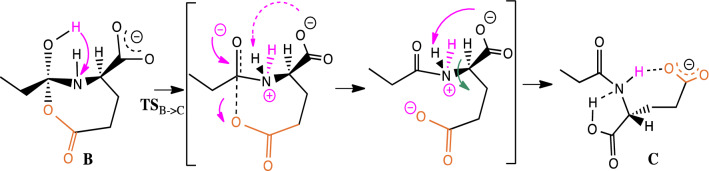
Formation of the **C** anion (deprotomer of the initial **A** anion) connected by **TS**_**B->C**_ as a dead end of this pathway

A transition state (**TS**_**A->B**_) leading to the cyclization into a tetrahedral intermediate **B** situated 40.8 kcal/mol above **A** and corresponding to an endothermic transformation by 36.1 kcal/mol could be located. This cyclization is facilitated by an assistance of the proton of the COOH group of the amino acid which stabilizes the produced alkoxide by its neutralization. In a second step, we explored the possibility of the C-N bond cleavage by proton migration from the OH group of tetrahedral intermediate to the neighbored nitrogen atom. This migration results in the creation of an ammonium group which should promote the anticipated cleavage to open the ring and to form an anhydride group (i.e., the **C’** intermediate). A transition state (**TS**_**B->C**_) for such a proton transfer was found (25.4 kcal/mol above **B**). However, the opened ring product **C** to which it connects is not the result of the C-N bond cleavage wished. Indeed, this proton transfer leads to the opening of the anhydride, immediately followed by the migration of the proton initially on -NH- groups which is captured by the -CO_2_**¯** group (Scheme 4).

**Fig. 3:**
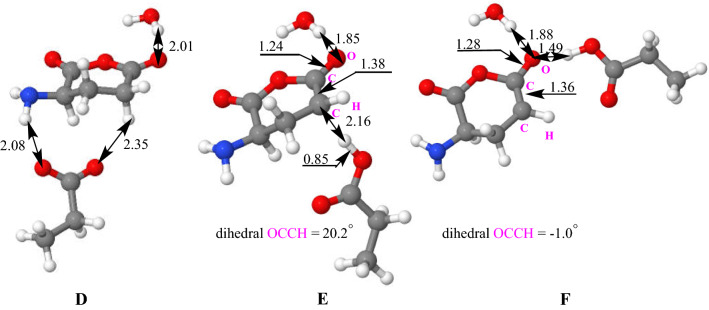
Structures **D**, **E** and **F**. Distances in Å

To strongly hinder protonation of the carboxylate group by an ammonium proton for favoring C-N cleavage and ring opening, a water molecule was introduced (**A’** + H_2_O) in calculations to check its effect on the re-orientation of reaction toward formation of the (**C’** + H_2_O) intermediate rather than that the **C** deprotomer. In a similar way, a first cyclization (via **TS**_**A’->B’**_ + H_2_O) leads to structure (**B’** + H_2_O) with a lower energy cost than in the absence of water (36.7 versus 40.8 kcal/mol). From the seven-membered ring tetrahedral (**B’** + H_2_O) intermediate, the proton transfers to the nitrogen atom triggers, this time, the rupture of the C-N bond to yield, through rearrangement process, the anhydride (**C’** + H_2_O) intermediate. This cleavage corresponds to an exothermic transformation by 8.1 kcal/mol with respect to the solvated (**B’** + H_2_O) form. At the end, to reach deprotonated fatty acid (ion [*lp*b + O]¯) from the (**C’** + H_2_O) intermediate, the carboxylic group must drive a nucleophilic attack at the adequate C-O bond of the anhydride group yielding ion/neutral complex (**D** intermediate stabilized by hydrogen bond) consisting in deprotonated fatty acid (herein propionic carboxylate, EtCOO¯) and the α amino glutaric anhydride neutral. This cyclization leading to **D** and the formation of the carboxylate ([*lp*b + O]¯) occurs via **TS**_**C’->D**_ with a barrier of 10.1 kcal/mol in an exothermic process of 3.0 kcal/mol. In the **D** intermediate (Fig. 3), the EtCOO^−^ group is favorably positioned to attract a mobile proton from the enolizable CH_2_ site of the carbonyl group. This deprotonation requires a cost of 9.1 kcal/mol to form an intermediate **E** located very slightly (1.4 kcal/mol) above the **TS**_**D->E**_. This is probably due to a persistent interaction between the OH group of the carboxylic acid and the CH that prevents the full formation of the enolate form. This is evidenced by the analysis of the value of the dihedral angle OC-CH in this structure which is 20.2 degrees, while it would expect a value very close to 0° for an enolate form. The system gains energy by migration of the acid towards the oxygen atom of the enolate by developing a hydrogen bond type interaction. In structure **F**, the enolate is this time completely formed, the previously discussed dihedral angle being now close to 0 degrees (− 1.0). Evolutions of the C–C and C–O distances between **E** and **F** are also logical.

Finally, the competitive dissociation of both the **D** and **F** intermediates are only desolvation steps (i.e., cleavage of hydrogen bond) characterized by level lower than the highest transition state (i.e**., TS**_**B’->C’**_) of these stepwise dissociations of the mono-hydrated model system of lipo-glutamic acid.

## Conclusion

In this study, LC-ESI-HRMS/MS performed in negative ion mode for the LpAA analysis has some advantages in terms of sensibility and specificity. Under low-energy collision-induced-dissociation (CID) conditions, [LpAA-H]¯ anion dissociate mainly into complementary product ion pair allowing to unambiguously qualify the two conjugated components of LpAA except for non-polar AA residues which present only one characteristic product ion. Using the peptide dissociation annotation, the complementary [*lp*c]¯ and [z-2H]¯ ion pair, respectively, corresponding to the fatty acid amidate and deaminated amino-acid carboxylate are abundant in the product ion spectra of [LpAA-H]¯. However, from deprotonated glutamate conjugates such as [C16Glu-H]¯, the [*lp*c]¯ and [z-2H]¯ anion pair disappears in favor to another diagnostic ion pair. Indeed, the complementary [*lp*b + O]¯ and [y-H_2_O]¯ product anions appears due to regeneration of fatty acid carboxylate and formation of dehydrated glutamate via a nucleophilic attack of amide group by one of the two carboxylic groups. This annotation was the one used for deprotonated peptide dissociation. Interestingly, dissociation of deprotonated C16GluMe mono methyl-ester located at the glutamic side chain leads to reduction of the [*lp*b + O]¯ ion abundance which evidences the strong regioselectivity of the δ carboxylic acid on the nucleophilic attack on amide linkage resulting in 7-membered ring with a tetrahedral reactive site. From this intermediate evolves toward a stepwise process via isomerization into ion–dipole intermediate from which through internal proton transfer and complex splitting yields in competition, the [*lp*b + O]¯ and [y-H_2_O]¯ product anions. The specific behavior of the glutamate residue allows it to be distinguished from other amino acid residues independently to the fatty acid amide chain length. Thanks to the modeling, the pertinence of the above proposed stepwise process mechanism leading to the complementary product ions especially the regeneration of the fatty acid carboxylate is confirmed. All these results allow to improve the identification of LpAA using mass spectrometry which is essential for the study of this low concentrated bacterial metabolites.

## Supplementary Information

Below is the link to the electronic supplementary material.

Supplementary file1 (DOCX 204 KB)

Supplementary file2 (DOCX 217 KB)

## Abbreviations

ACN: Acetonitrile
CID: Collision-induced dissociation
DCM: Dichloromethane
DFT: Density functional theory
EcN: Escherichia coli
ESI: Electrospray
GABA: γ- Aminobutyric acid
HOBt: 1-Hydroxybenzotriazole hydrate
Fmoc: 9-Fluorenylmethoxycarbonyl
LC-HRMS: Liquid-chromatography high-resolution mass spectrometry
LpAA: Lipoaminoacids
MeOH: Methanol
NMM: N-methylmorpholine
NMP: N-methyl-2-pyrrolidone
RF: Radio-frequency
TFA: Trifluoroacetic acid
TIPS: Triisopropylsilane
ZPE: Zero point energy
