# Supplementary material for: Identification of bacterial lipo-amino acids: origin of regenerated fatty acid carboxylate from dissociation of lipo-glutamate anion
Source: Amino Acids. 2022 Jan 25;54(2):241–50. doi: 10.1007/s00726-021-03109-1 (PMC8894203; doi:10.1007/s00726-021-03109-1)
Supplement: Supplementary file 1 — Supplementary file1 (DOCX 204 KB) [file 726_2021_3109_MOESM1_ESM.docx]

**Supplementary Informations**

**New lipoamino acid from *E.Coli* Bacteria: origin of regenerated fatty acid carboxylate from dissociation of lipo-glutamate anion**

Amandine Hueber ^a,b^, Yves Gimbert^c,d^, Geoffrey Langevin^e^, Nicolas Cenac^b^, Justine Bertrand-Michel^a,f^, Jean-Claude Tabet^a,d, g^

^a^. MetaboHUB-MetaToul, National Infrastructure of Metabolomics and Fluxomics, Toulouse, F-31077, France

^b^. IRSD, Université de Toulouse, INSERM, INRA, INPENVT, Université de Toulouse 3 Paul Sabatier, F-31024 Toulouse, France

^c^. Département de chimie Moléculaire, UMR CNRS, 5250, Université Grenoble Alpes, F-38050 Grenoble, France

^d^. Université, CNRS, Institut Parisien de Chimie Moléculaire (UMR 8232), 4 place Jussieu, F-75005 Paris, France

^e^. Institut des Biomolécules Max Mousseron, UMR 5247 CNRS, Université de Montpellier-ENSCM, F-34093 Montpellier, France

^f^. I2MC, Université de Toulouse, Inserm, Université Toulouse 3 Paul Sabatier, F-31432 Toulouse, France

^g^. Université Paris-Saclay, CEA, INRAE, Département Médicaments et Technologies pour la Santé, F-91191 Gif-sur-Yvette, France

*Corresponding author : Justine Bertrand-Michel, justine.bertrand-michel@inserm.fr


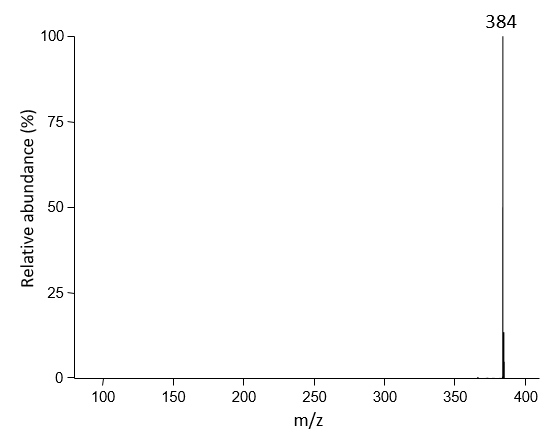


**Figure S1**. Negative ESI mass spectrum of the C16Glu standard (Mw=385 u)

[C12Asn-H]^-^
m/z 313; E_lab_ = 17eV

100

200

300

0

25

50

75

100

313

295

198

96

114

131

269

251

224

100

200

300

0

25

50

75

100

312

130

198

268

**A)**

**B)**

[C12Leu-H]^-^
m/z 312; E_lab_ = 17eV

Relative abundance (%)

Relative abundance (%)

m/z

m/z

181

**Figure S2**. Product ion spectra of A) [C12Asn-H]^-^ (m/z 313), at E_Lab_ = 17 eV (a), and B) [C12Leu-H]ˉ (m/z 312) at E_Lab_ = 17 eV (b)


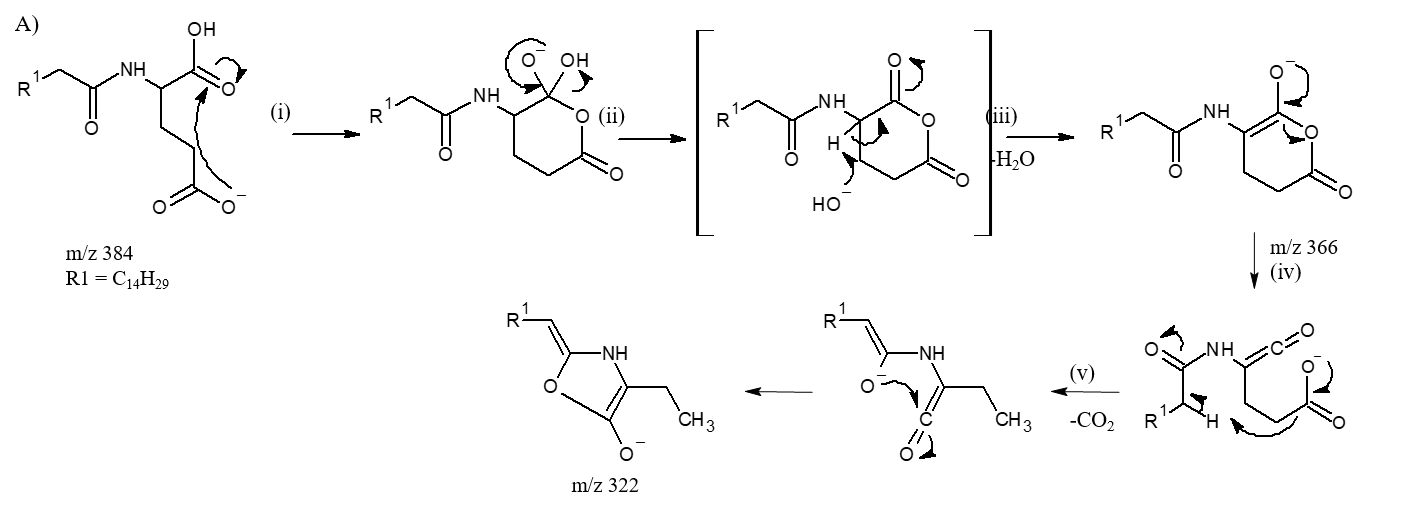

(v)

(v)

-CO_2_

-CO_2_

m/z 384

m/z 340

m/z 296

R^1^ = C_14_H_29_

**Scheme S1**. Possible interpretations of the small size losses from competitive and consecutive dissociations of the [C16Glu-H]¯ anion: (a) stepwise processes (i) nucleophilic attack yielding cyclisation and (ii) ion-dipole intermediate resulting in (iii) the H_2_O release (ion m/z 366) followed consecutively (iv)+(v) by the CO_2_ loss (ion m/z 322); and (b) the direct loss of CO_2_ (v) (m/z 340), competitive to the stepwise water loss (a), followed consecutively by a second loss of CO_2_ (v) (m/z 296).


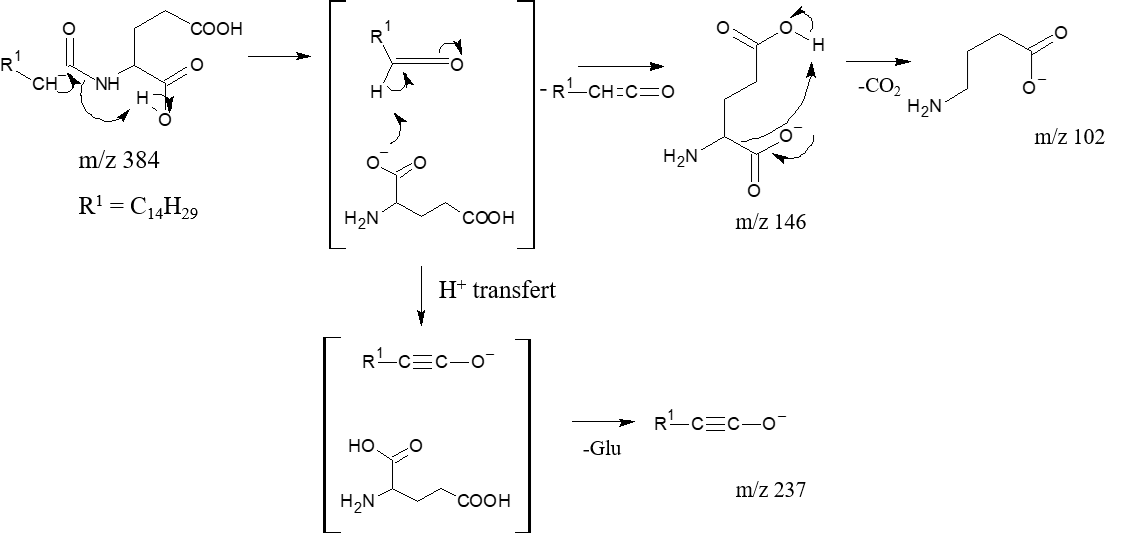


**Scheme S2** : Possible interpretation of the complementary product m/z 146 and m/z 237 ion pair *via* molecular species isomerization into ion-dipole intermediate yielding either by direct partner splitting, product m/z 146 ion (able to release consecutively CO_2_ into m/z 102) or by internal proton transfer and isomerized intermediate splitting in the ynolate m/z 237 ion.

**Table S1**. Elemental compositions and relative (and absolute) abundances of product ion displayed in CID spectra of deprotonated C16Glu (m/z 384), C16GluMe (m/z 398), and C12Leu (m/z 312) molecules recorded at 17 eV and 30 eV (annotation concern the amide bond linkage)

| **Precursor ions** | **Product ions** | | | | | | Collision energy  E_lab_ = 17 eV | | Collision energy E_lab_ = 30 eV | |
| --- | --- | --- | --- | --- | --- | --- | --- | --- | --- | --- |
|  | m/z values (experimental) | Elemental composition | m/z values (calculated) | Error (ppm) | Formal loss | Product ions Annotation | Relative abondance | Absolute abundances | Relative abondance | Absolute abundances |
| **[C16Glu-H]ˉ** | 384.2756 | C_21_H_38_NO_5_ | 384.2755 | 0.2 | H |  | 35.7 | 16414096 | 0.08 | 15577 |
|  | 366.2664 | C_21_H_36_NO_4_ | 366.2649 | 3.8 | H_2_O |  | 10.4 | 4780996 | 0.02 | 4858 |
|  | 340.2848 | C_20_H_38_NO_3_ | 340.2857 | 2.7 | CO_2_ |  | 18.8 | 8640096 | 2.5 | 467789 |
|  | 322.2751 | C_22_H_36_NO_2_ | 322.2746 | 0.1 | CH_2_O_3_ |  | 0.7 | 344643 | 1.8 | 332035 |
|  | 296.2957 | C_19_H_38_NO | 296.2958 | 0.6 | C_2_O_4_ |  | 1.1 | 529566 | 20.9 | 3919886 |
|  | 255.2321 | C_16_H_31_O_2_ | 255.2329 | 3.3 | C_5_H_7_NO_3_ | [*Lp*b+O]ˉ | 20.5 | 9450200 | 33.2 | 6221260 |
|  | 254.2483 | C_16_H_32_NO | 254.2489 | 2.5 | C_5_H_6_O_4_ | cˉ | 0.3 | 140354 | 6.5 | 1223542 |
|  | 237.2213 | C_16_H_29_O | 237.2218 | 4.5 | C_4_H_9_NO_4_ | [*Lp*b-2H]ˉ- | 0.04 | 18665 | 1.7 | 324200 |
|  | 146.0454 | C_5_H_8_NO_4_ | 146.0458 | 3.3 | C_16_H_30_O | yˉ | 1.4 | 621838 | 3.0 | 563895 |
|  | 128.0350 | C_5_H_6_NO_3_ | 128.0353 | 2.4 | C_16_H_32_O_2_ | [z-2H]ˉ | 10.3 | 4768896 | 16.9 | 3175950 |
|  | 102.0565 | C_4_H_8_NO_2_ | 102.0555 | 4.3 | C_17_H_30_O_3_ | [y-H_2_O]ˉ | 0.6 | 291877 | 12.9 | 2451976 |
|  | 84.0455 | C_4_H_6_NO | 84.0454 | 0.2 | C_17_H_32_O_4_ | [y-CO_2_]ˉ | 0.01 | 5925 | 0.4 | 83752 |
| **[C16GluMe-H]ˉ** | 398.2908 | C_22_H_40_NO_5_ | 398.2911 | 1.0 | H |  | 11.2 | 1960016 | 0 | 0 |
|  | 380.2818 | C_22_H_38_NO_4_ | 380.2806 | 3.0 | H_2_O |  | 0.04 | 8075 | 0 | 0 |
|  | 366.2642 | C_21_H_36_NO_4_ | 366.2649 | 2.1 | CH_4_O |  | 43.8 | 7638936 | 0 | 0 |
|  | 338.2352 | C_19_H_32_NO_4_ | 338.2336 | 4.8 | C_3_H_8_O |  | 0.06 | 10589 | 0 | 0 |
|  | 322.2741 | C_20_H_36_NO_2_ | 322.2751 | 3.3 | C_2_H_4_O_3_ |  | 1.12 | 195736 | 0.7 | 14267 |
|  | 312.2532 | C_18_H_34_NO_3_ | 312.2544 | 3.9 | C_4_H_6_O_2_ |  | 0.2 | 33988 | 0 | 0 |
|  | 294.2423 | C_18_H_32_NO_2_ | 294.2438 | 5.2 | C_4_H_8_O_3_ |  | 0.2 | 28252 | 2.0 | 39971 |
|  | 280.2632 | C_18_H_34_NO | 280.2645 | 0.3 | C_4_H_6_O_4_ |  | 0.7 | 114670 | 8.4 | 169650 |
|  | 268.2643 | C_17_H_34_NO | 268.2645 | 1.0 | C_5_H_6_O_4_ |  | 0.03 | 6654 | 0 | 0 |
|  | 255.2324 | C_16_H_31_O_2_ | 255.2329 | 2.2 | C_6_H_9_NO_3_ | [*Lp*b+O]ˉ- | 0.9 | 167446 | 9.3 | 187801 |
|  | 254.2484 | C_16_H_32_NO | 254.2489 | 2.1 | C_6_H_8_NO_4_ | cˉ | 0.001 | 274 | 0 | 0 |
|  | 237.2210 | C_16_H_29_O | 237.2218 | 5.8 | C_6_H_11_O_4_ | [*Lp*b-2H]ˉ- | 0.02 | 4894 | 1.5 | 30027 |
|  | 160.0621 | C_6_H_10_NO_4_ | 160.0615 | 3.5 | C_16_H_30_O | y¯ | 1.0 | 182477 | 0.4 | 7798 |
|  | 128.0343 | C_5_H_6_NO_3_ | 128.0353 | 3.5 | C_17_H_34_O_2_ | [y-MeOH]ˉ- | 40.7 | 7494856 | 77.8 | 1567510 |
|  | 84.0450 | C_4_H_6_NO | 84.0454 | 5.8 | C_18_H_34_O_4_ | [y-MeOH-CO_2_]ˉ- | 0.01 | 2414 | 0.2 | 4604 |
| **[C12Leu-H]ˉ** | 312.2539 | C_18_H_34_NO_3_ | 312.2544 | 1.7 | H |  | 49.5 | 6934268 | 0 | 0 |
|  | 268.2628 | C_17_H_34_NO | 268.2645 | 1.3 | CO_2_ |  | 8.4 | 1318843 | 2.5 | 87115 |
|  | 198.1847 | C_12_H_24_NO | 198.1863 | 1.3 | C_6_H_10_O_2_ | c¯ | 0.7 | 129572 | 1.8 | 63622 |
|  | 130.0871 | C_6_H_12_NO_2_ | 130.0873 | 1.9 | C_12_H_22_O | y¯ | 41.3 | 5794856 | 95.6 | 3314254 |

**Information S1**:

Modelling has been performed at B3LYP/6-31+G(d,p) (Stephens, 1994) level using G16 package (Frisch , 2016) the nature of minima identified by the nature of their frequencies.

**A** *E(ZPE)= -742.942528*

6 0.29636 1.11206 0.38202

1 0.52596 0.96883 1.452

6 1.36572 2.11024 -0.13349

8 1.09597 3.2276 -0.53296

8 2.63017 1.68341 -0.05758

1 2.65739 0.72647 0.27111

6 -1.12342 1.68048 0.24406

1 -1.12307 2.65189 0.74837

1 -1.31747 1.88897 -0.81363

6 -2.2486 0.79607 0.82348

1 -1.90299 0.30237 1.74497

1 -3.0908 1.42818 1.12093

6 -2.83866 -0.31409 -0.10013

8 -2.03424 -0.85547 -0.94433

8 -4.03727 -0.60244 0.06765

7 0.3611 -0.19975 -0.27344

1 -0.60478 -0.52633 -0.6613

6 1.3364 -1.08354 -0.06042

8 2.40699 -0.81089 0.5517

6 1.11742 -2.4799 -0.62321

1 1.82791 -2.60501 -1.45108

1 0.10711 -2.5591 -1.03487

6 1.36572 -3.56874 0.4303

1 2.36552 -3.46752 0.8619

1 0.63415 -3.49968 1.24283

1 1.27468 -4.56398 -0.01863

**TS**_A->B_  *E(ZPE)=* *-742,877372*

6 -1.314794 0.227540 0.058753

1 -0.873724 0.315579 -0.939059

6 -2.524797 -0.707286 -0.191687

8 -3.676303 -0.369566 0.134119

8 -2.130462 -1.780711 -0.773727

1 -0.531503 -1.753617 -0.933405

6 -1.415873 1.658374 0.560071

1 -1.998233 2.248970 -0.154228

1 -1.941891 1.720220 1.521492

6 0.024529 2.237548 0.697065

1 -0.001536 3.329261 0.667903

1 0.443506 1.964789 1.675407

6 1.066009 1.825805 -0.378936

8 1.464710 0.568916 -0.523087

8 1.567887 2.714689 -1.066542

7 -0.324455 -0.499404 0.892434

1 -0.108346 -0.036402 1.765000

6 0.802237 -0.857444 0.163638

8 0.500459 -1.628033 -0.894160

6 1.990088 -1.349469 0.978306

1 1.632789 -2.231197 1.526181

1 2.253369 -0.582862 1.717205

6 3.212425 -1.716675 0.133885

1 2.947595 -2.475018 -0.608121

1 3.590712 -0.839853 -0.396948

1 4.009176 -2.116760 0.772138

**B** *E(ZPE)=-742.884861*

6 -1.393647 0.042026 -0.216064

1 -1.293327 -0.057944 -1.313138

6 -2.713054 -0.748606 0.149827

8 -2.536670 -1.828354 0.777114

8 -3.775066 -0.219277 -0.254166

1 0.167790 -1.410239 -1.784689

6 -1.504003 1.533686 0.116713

1 -2.418881 1.929261 -0.333063

1 -1.597204 1.628872 1.205117

6 -0.291361 2.367362 -0.362103

1 -0.317127 2.438369 -1.457515

1 -0.327611 3.377671 0.056610

6 1.036751 1.743476 0.011125

8 1.338444 0.626793 -0.744062

8 1.823102 2.199842 0.813205

7 -0.251701 -0.549319 0.495768

1 -0.594647 -1.430637 0.894186

6 0.992374 -0.687965 -0.185677

8 0.993600 -1.558008 -1.302088

6 2.091245 -1.170535 0.766296

1 1.745301 -2.116964 1.200175

1 2.152154 -0.439808 1.577361

6 3.460481 -1.354184 0.107867

1 3.421271 -2.106195 -0.685504

1 3.805014 -0.412451 -0.330195

1 4.199184 -1.674240 0.852368

**TS**_B->C_ *E(ZPE)=* *-742.844254*

6 -1.501893 0.269480 -0.386423

1 -1.627205 0.728737 -1.371816

6 -2.903004 -0.281548 0.101192

8 -2.860222 -1.426543 0.634792

8 -3.863831 0.499116 -0.062716

1 -0.147547 -1.567143 -1.477759

6 -1.015260 1.316987 0.620783

1 -1.871708 1.968307 0.824016

1 -0.769199 0.822048 1.569250

6 0.144268 2.206607 0.156421

1 -0.164086 2.723159 -0.765236

1 0.352214 2.987843 0.894246

6 1.480578 1.568724 -0.190626

8 1.479984 0.393917 -0.862283

8 2.527845 2.168459 -0.018221

7 -0.596233 -0.896348 -0.496358

1 -1.038815 -1.583955 0.145315

6 0.916510 -0.914626 -0.395785

8 1.128170 -1.829171 -1.350903

6 1.495360 -1.227454 0.987438

1 0.983500 -2.129264 1.343688

1 1.247795 -0.422141 1.690759

6 3.009062 -1.460633 0.952249

1 3.246956 -2.256645 0.241034

1 3.537468 -0.555462 0.640273

1 3.374956 -1.752502 1.943885

**C** *E(ZPE)=* *-742.911690*

6 0.174305 0.812153 -0.224058

1 0.953024 1.144653 -0.924657

6 -0.832844 1.964769 -0.238044

8 -1.616581 1.986772 -1.349439

8 -0.910701 2.848710 0.586268

1 0.431541 -0.894573 -1.300163

6 0.890409 0.553840 1.120003

1 0.624870 1.354871 1.819357

1 0.514994 -0.385847 1.542553

6 2.423641 0.476372 0.986057

1 2.800321 1.464402 0.678593

1 2.865265 0.273850 1.967218

6 3.011269 -0.559706 -0.029988

8 4.125165 -1.039758 0.271126

8 2.331256 -0.774521 -1.086763

7 -0.367221 -0.382838 -0.895611

1 -1.422279 1.164658 -1.843219

6 -1.372051 -1.197168 -0.407190

8 -1.519395 -2.340759 -0.834128

6 -2.348100 -0.603944 0.613831

1 -2.918344 0.193628 0.120270

1 -1.788425 -0.113185 1.417742

6 -3.302965 -1.649399 1.192386

1 -3.876950 -2.136720 0.399955

1 -2.753731 -2.433094 1.722647

1 -3.999668 -1.177838 1.894479

**A’**+H_2_O *E(ZPE)= -819.369259*

6 -0.451947 -0.540910 0.508094

1 -0.438826 -0.272717 1.578931

6 -1.946511 -0.517222 0.111671

8 -2.564771 -1.521178 -0.212912

8 -2.552179 0.663878 0.201821

1 -1.876585 1.393118 0.459064

6 0.163579 -1.937158 0.334121

1 -0.469990 -2.633610 0.891917

1 0.082735 -2.229008 -0.718624

6 1.626084 -2.073598 0.810213

1 1.781597 -1.478318 1.723216

1 1.819656 -3.112435 1.093051

6 2.749051 -1.676515 -0.195766

8 2.484422 -0.716556 -1.012695

8 3.819657 -2.301664 -0.118602

7 0.358910 0.443623 -0.218222

1 1.272503 0.001922 -0.656499

6 0.261526 1.758287 -0.034174

8 -0.676004 2.308885 0.615266

6 1.339367 2.616234 -0.676257

1 0.859712 3.169372 -1.494602

1 2.109916 1.974978 -1.113566

6 1.953410 3.611003 0.319014

1 1.176709 4.231106 0.775189

1 2.485656 3.085654 1.119518

1 2.672027 4.263603 -0.188492

8 -5.336824 -0.684213 -0.500763

1 -5.066129 0.224221 -0.313938

1 -4.483119 -1.157196 -0.466025

**TS**_A’->B’_  *E(ZPE)=* *-819.310747*

6 0.668665 -0.739843 0.142713

1 0.278643 -0.631674 -0.873496

6 2.141433 -0.305986 -0.003067

8 3.076017 -1.042520 0.368421

8 2.215570 0.851149 -0.555598

1 0.702748 1.424961 -0.807815

6 0.227925 -2.125386 0.584709

1 0.606737 -2.860996 -0.131816

1 0.648527 -2.394038 1.562077

6 -1.328464 -2.170073 0.634294

1 -1.673088 -3.205471 0.587828

1 -1.681135 -1.770603 1.595763

6 -2.098322 -1.419889 -0.490473

8 -2.021521 -0.110547 -0.609476

8 -2.821238 -2.089274 -1.232825

7 -0.036121 0.274476 0.966429

1 -0.458228 -0.100647 1.804983

6 -0.889323 1.057987 0.223667

8 -0.295466 1.683845 -0.790563

6 -1.920414 1.876133 0.974594

1 -1.349706 2.572356 1.604812

1 -2.479641 1.208032 1.638716

6 -2.878134 2.653440 0.068778

1 -2.322734 3.325646 -0.591061

1 -3.455597 1.965451 -0.552236

1 -3.566598 3.252976 0.675239

8 5.144380 0.838388 -0.362178

1 4.314634 1.278745 -0.616158

1 4.766154 0.007311 -0.015373

**B’**+H_2_O *E(ZPE)=* *-819.320355*

6 0.844882 0.445772 0.308667

1 0.710277 0.368446 1.403136

6 2.316355 -0.030441 0.044997

8 2.444017 -1.135455 -0.551659

8 3.215472 0.733148 0.483118

1 -0.471673 -1.247752 1.901513

6 0.641136 1.912275 -0.092672

1 1.417074 2.520220 0.380278

1 0.774208 1.984042 -1.178416

6 -0.751367 2.470224 0.285599

1 -0.803772 2.595576 1.375007

1 -0.917272 3.442211 -0.187981

6 -1.882608 1.545414 -0.110658

8 -1.961748 0.422117 0.692359

8 -2.704837 1.772073 -0.970812

7 -0.094112 -0.414837 -0.422881

1 0.444570 -1.212232 -0.772138

6 -1.314343 -0.802403 0.209637

8 -1.185196 -1.613927 1.360116

6 -2.224946 -1.549057 -0.769991

1 -1.657239 -2.411448 -1.140871

1 -2.396567 -0.879492 -1.616982

6 -3.555933 -2.005601 -0.168717

1 -3.398590 -2.705403 0.656926

1 -4.119398 -1.148430 0.212106

1 -4.165534 -2.502374 -0.932511

8 5.358419 -0.953852 -0.360065

1 4.559502 -1.430682 -0.647216

1 4.915120 -0.178746 0.041696

**TS**_B’->C’_ *E(ZPE)=* *-819.277877*

6 -0.926645 0.479311 -0.528001

1 -0.895750 0.971506 -1.504419

6 -2.414502 0.095242 -0.207347

8 -2.580270 -1.045567 0.312447

8 -3.264189 0.981310 -0.460990

1 0.278912 -1.465894 -1.561710

6 -0.425220 1.440203 0.558445

1 -1.222044 2.174216 0.716630

1 -0.315183 0.892767 1.503377

6 0.855572 2.214815 0.224223

1 0.676479 2.800742 -0.689883

1 1.087160 2.935368 1.014425

6 2.135634 1.444091 -0.057414

8 2.047610 0.313535 -0.801008

8 3.226425 1.902252 0.230116

7 -0.152341 -0.780789 -0.590415

1 -0.688065 -1.430237 0.010356

6 1.350881 -0.950913 -0.411316

8 1.522933 -1.852944 -1.382735

6 1.812844 -1.357846 0.990947

1 1.194857 -2.213392 1.288582

1 1.606452 -0.550945 1.705817

6 3.295083 -1.742934 1.030099

1 3.492019 -2.538728 0.306394

1 3.928896 -0.888037 0.778960

1 3.571171 -2.098707 2.029595

8 -5.459368 -0.541739 0.606811

1 -4.688238 -1.125342 0.714445

1 -5.007280 0.215619 0.185387

**C’**+H_2_O *E(ZPE)=* *-819.333292*

6 1.491043 -0.411369 -0.549449

1 1.493475 -0.981263 -1.487190

6 2.953124 0.083772 -0.295894

8 3.074313 1.278113 0.099191

8 3.857898 -0.773319 -0.487785

1 0.560232 1.060556 -1.611073

6 1.072637 -1.360495 0.583297

1 1.812757 -2.163378 0.652620

1 1.082544 -0.821714 1.538786

6 -0.321873 -1.979272 0.365564

1 -0.403970 -2.410207 -0.635992

1 -0.476013 -2.787473 1.093018

6 -1.454899 -1.010000 0.592521

8 -2.352973 -1.013615 -0.468870

8 -1.680287 -0.401114 1.613106

7 0.510824 0.687978 -0.664401

1 0.899943 1.437649 -0.083286

6 -3.329308 -0.034512 -0.603610

8 -4.405643 -0.371388 -1.036939

6 -2.936420 1.400808 -0.320783

1 -3.121788 1.934363 -1.261871

1 -1.870875 1.465443 -0.098184

6 -3.789082 2.024809 0.795703

1 -4.856218 1.941196 0.567246

1 -3.589877 1.528721 1.749062

1 -3.540353 3.085700 0.902776

8 5.960728 1.019334 0.236073

1 5.136277 1.529827 0.339835

1 5.554470 0.178188 -0.057245

**TS**_C’->D_ *E(ZPE)=* *-819.277877*

6 2.197781 -0.872935 0.192059

1 1.482023 -1.689802 0.053869

6 1.486674 0.199494 1.026726

8 1.771250 0.438616 2.188121

8 0.541131 0.933917 0.410522

1 3.143953 -1.821703 1.720526

6 2.515514 -0.274197 -1.185050

1 3.225627 0.553946 -1.044507

1 3.029972 -1.032093 -1.783832

6 1.251504 0.229914 -1.903106

1 1.483444 1.099518 -2.526612

1 0.834861 -0.543609 -2.550226

6 0.107493 0.675833 -0.986944

8 -0.562064 -1.041211 -0.730928

8 -0.747446 1.470051 -1.373064

7 3.401912 -1.401831 0.830288

1 4.020802 -0.629507 1.073699

6 -1.733459 -1.362066 -0.237046

8 -2.136620 -2.531247 -0.226647

6 -2.590813 -0.236097 0.345948

1 -2.718229 0.525667 -0.429785

1 -2.006191 0.260199 1.130104

6 -3.940173 -0.705339 0.890685

1 -4.536982 -1.182852 0.106869

1 -3.813018 -1.443161 1.689251

1 -4.507322 0.145105 1.289537

8 -1.066410 3.659605 0.447701

1 -0.539865 3.225195 1.131465

1 -1.078323 2.980397 -0.257643

**D** *E(ZPE)=* *-819.338176*

6 0.211522 1.064775 0.294939

1 -0.344399 0.476787 -0.466044

6 1.584389 1.277426 -0.267803

8 2.014175 2.275604 -0.787749

8 2.462317 0.171922 -0.233031

1 -1.457061 2.112089 0.543353

6 0.268449 0.144173 1.519247

1 0.838124 0.618546 2.328784

1 -0.757486 -0.008577 1.860596

6 0.873466 -1.190391 1.085542

1 1.013826 -1.900827 1.904231

1 0.178732 -1.643546 0.360297

6 2.201975 -1.026600 0.404913

8 -1.865343 -1.056837 -0.631666

8 3.078302 -1.865723 0.360426

7 -0.454546 2.326286 0.598268

1 -0.238398 3.006127 -0.127226

6 -2.915883 -0.412921 -0.333145

8 -2.977922 0.736579 0.188286

6 -4.250651 -1.135994 -0.648496

1 -4.231709 -2.094413 -0.111846

1 -4.229137 -1.399255 -1.714516

6 -5.520615 -0.352912 -0.309594

1 -5.552278 -0.101781 0.755449

1 -5.553243 0.594261 -0.857595

1 -6.422319 -0.930072 -0.558723

8 5.466410 -0.764826 -0.999829

1 5.034676 0.076513 -1.197188

1 4.763066 -1.268943 -0.552566

**TS**_D->E_ *E(ZPE)=* *-819.323584*

6 0.556785 1.685186 0.129827

1 -0.257211 1.252720 -0.475671

6 1.836407 1.191096 -0.520372

8 2.511557 1.868126 -1.275829

8 2.199005 -0.089614 -0.281918

1 -0.436582 3.466618 0.081478

6 0.404254 1.100430 1.536609

1 1.158270 1.564887 2.192819

1 -0.579718 1.398014 1.916593

6 0.505221 -0.413147 1.463441

1 0.346587 -0.959004 2.394449

1 -0.788484 -0.905912 0.774524

6 1.567027 -0.966281 0.713282

8 -1.761212 -1.366316 0.399871

8 2.045875 -2.096649 0.705100

7 0.528180 3.151009 0.121246

1 1.011991 3.489601 -0.707561

6 -2.684370 -0.524389 0.005207

8 -2.590106 0.705044 0.010056

6 -3.947976 -1.234295 -0.488807

1 -4.283774 -1.901153 0.315371

1 -3.646554 -1.896917 -1.310085

6 -5.067043 -0.289856 -0.926227

1 -5.378425 0.358748 -0.101569

1 -4.734517 0.362567 -1.739201

1 -5.940623 -0.856425 -1.270797

8 4.236933 -2.375349 -1.119427

1 4.170971 -1.461984 -1.427222

1 3.502577 -2.423218 -0.472419

**E** *E(ZPE)=* *-819.321286*

6 0.641561 1.708415 0.105284

1 -0.182333 1.277465 -0.487995

6 1.908815 1.157169 -0.529925

8 2.602072 1.802993 -1.300055

8 2.227257 -0.121099 -0.260343

1 -0.298566 3.518682 -0.029356

6 0.477493 1.173497 1.531130

1 1.238456 1.653953 2.169402

1 -0.499664 1.505004 1.902807

6 0.557572 -0.337754 1.516656

1 0.311032 -0.873142 2.432445

1 -0.914464 -0.898002 0.759678

6 1.548029 -0.965827 0.758765

8 -1.814035 -1.344350 0.433339

8 1.964300 -2.125667 0.737363

7 0.653480 3.173474 0.047237

1 1.172205 3.468534 -0.777265

6 -2.725462 -0.485098 0.011202

8 -2.598748 0.734753 -0.006464

6 -3.989160 -1.192757 -0.470874

1 -4.329255 -1.847343 0.341261

1 -3.691554 -1.867195 -1.283810

6 -5.098393 -0.242795 -0.919920

1 -5.406648 0.416186 -0.102470

1 -4.758928 0.397642 -1.739288

1 -5.974983 -0.806392 -1.259930

8 4.050388 -2.555148 -1.147515

1 4.049726 -1.637041 -1.448236

1 3.338022 -2.543621 -0.472411

**F** *E(ZPE)=* *-819.326603*

6 3.019860 1.261705 -0.365224

1 2.766462 1.183381 -1.438705

6 3.134528 -0.188317 0.099414

8 4.209164 -0.738470 0.287275

8 1.993762 -0.867724 0.257070

1 4.462918 2.643606 -0.853722

6 1.828301 1.936018 0.326802

1 2.100051 2.100877 1.383388

1 1.690666 2.934161 -0.108747

6 0.592837 1.098403 0.136764

1 -0.384832 1.550372 0.017937

6 0.664489 -0.253726 0.082230

8 -2.703595 -0.995503 0.244730

8 -0.214340 -1.164937 -0.069777

7 4.292487 1.949221 -0.133752

1 5.048446 1.267563 -0.148874

6 -3.391200 0.097573 -0.027383

8 -2.924927 1.157813 -0.427705

6 -4.885962 -0.104874 0.219150

1 -5.004611 -0.433841 1.259341

1 -5.205769 -0.956524 -0.394776

6 -5.734319 1.132635 -0.068972

1 -5.423581 1.975524 0.555791

1 -5.625959 1.448744 -1.110905

1 -6.794472 0.929421 0.124049

8 0.938595 -3.749643 -0.350546

1 1.819156 -3.447334 -0.093159

1 0.416810 -2.920261 -0.286154

1 -1.673165 -0.929041 0.085747

**Bibliography**

Stephens,P.J., Devlin,F.J., Cablowski, C.F., et Frish,C.F. 1994. « Ab initio Calculation of vibrational absorption and circular dichroism spectra using dendity functional force fields ». *The journal of Physical Chemistry*, American Chemical Scociety.

Frisch, M. J.; Trucks, G. W.; Schlegel, H. B.; Scuseria, G. E.; Robb, M. A.; Cheeseman, J. R.; Scalmani, G.; Barone, V.; Petersson, G. A.; Nakatsuji, H.; Li, X.; Caricato, M.; Marenich, A. V.; Bloino, J.; Janesko, B. G.; Gomperts, R.; Mennucci, B.; Hratchian, H. P.; Ortiz, J. V.; Izmaylov, A. F.; Sonnenberg, J. L.; Williams-Young, D.; Ding, F.; Lipparini, F.; Egidi, F.; Goings, J.; Peng, B.; Petrone, A.; Henderson, T.; Ranasinghe, D.; Zakrzewski, V. G.; Gao, J.; Rega, N.; Zheng, G.; Liang, W.; Hada, M.; Ehara, M.; Toyota, K.; Fukuda, R.; Hasegawa, J.; Ishida, M.; Nakajima, T.; Honda, Y.; Kitao, O.; Nakai, H.; Vreven, T.; Throssell, K.; Montgomery, J. A., Jr.; Peralta, J. E.; Ogliaro, F.; Bearpark, M. J.; Heyd, J. J.; Brothers, E. N.; Kudin, K. N.; Staroverov, V. N.; Keith, T. A.; Kobayashi, R.; Normand, J.; Raghavachari, K.; Rendell, A. P.; Burant, J. C.; Iyengar, S. S.; Tomasi, J.; Cossi, M.; Millam, J. M.; Klene, M.; Adamo, C.; Cammi, R.; Ochterski, J. W.; Martin, R. L.; Morokuma, K.; Farkas, O.; Foresman, J. B.; Fox, D. J. Gaussian 16, Revision C.01 Gaussian, Inc., Wallingford CT, 2016.
